# Supplementary material for: Content-rich biological network constructed by mining PubMed abstracts
Source: BMC Bioinformatics. 2004 Oct 8;5:147. doi: 10.1186/1471-2105-5-147 (PMC528731; doi:10.1186/1471-2105-5-147)
Supplement: Additional File 5 — The original Chilibot query results of the term "long-term potentiation (LTP)" and 22 other terms, limiting the latest references analyzed to the years 1990, 1995, 2000, and 2004. [file 1471-2105-5-147-S5.bz2 › chilibotAdditionalFile5/ltp2000/html/PKA_ZIF268.html]

 


 **PKA** and **ZIF268** 
  
Found 5 abstracts in PubMed,  **5 abstracts were retrieved and analyzed**.  


---

 Search Google  |
 PDF files only 
|  EDU domain only 

---

- Brain Res, 2000   **Degree of immediate early gene induction in striatum by eticlopride determines sensitivity to N methyl D aspartate receptor blockade.** .
  Cortical afferents excite striatal efferent neurons through activation of N methyl D aspartate NMDA receptors, which can be modulated by D2 dopamine receptors.
  It is suggested that activation of **PKA** by D2 receptor blockade leads to NMDA receptor phosphorylation in the dendrites or phosphorylation of transcription factors in the nucleus.
  Thus, the levels and cellular localization of activated **PKA** may determine if D2 antagonist mediated gene expression is dependent on NMDA receptor activation.
  We have previously demonstrated that NMDA receptor antagonists block gene expression induced by a high dose of eticlopride in medial and central but not lateral striatum.
  Here, we examined the effects of NMDA receptor antagonists on striatal gene expression after administration of a low dose of eticlopride.
  The results showed that NMDA receptor antagonists blocked gene induction by eticlopride throughout striatum.
  Less **PKA** activation by the low dose of eticlopride might explain why the expression was more sensitive in the lateral striatum to NMDA receptor blockade than in our previous study.
  To increase levels of **PKA** activation to the extent that NMDA receptor blockade would have less effect on eticlopride mediated gene induction in all regions of striatum, we administered the phosphodiesterase inhibitor IBMX to animals treated with eticlopride.
  The combined administration of IBMX and eticlopride induced gene expression that was only partially attenuated c fos or unaffected **zif268** by NMDA receptor blockade.
  These data support the suggestion that the degree of second messenger activation by D2 receptor blockade determines whether D2 dopamine receptor antagonist mediated gene expression is dependent on NMDA receptor activation.

  - J Biol Chem, 1999   **Identification of glucagon like peptide 2 GLP 2 activated signaling pathways in baby hamster kidney fibroblasts expressing the rat GLP 2 receptor.** .
    Glucagon like peptide 2 GLP 2 promotes the expansion of the intestinal epithelium through stimulation of the GLP 2 receptor, a recently identified member of the glucagon secretin G protein coupled receptor superfamily.
    Although activation of G protein coupled receptors may lead to stimulation of cell growth, the mechanisms transducing the GLP 2 signal to mitogenic proliferation remain unknown.
    We now report studies of GLP 2R signaling in baby hamster kidney BHK cells expressing a transfected rat GLP 2 receptor BHK GLP 2R cells .
    GLP 2, but not glucagon or GLP 1, increased the levels of cAMP and activated both cAMP response element and AP 1 dependent transcriptional activity in a dose dependent manner.
    The activation of AP 1 luciferase activity was protein kinase A **PKA** dependent and markedly diminished in the presence of a dominant negative inhibitor of **PKA**.
    Although GLP 2 stimulated the expression of c fos, c jun, junB, and **zif268**, and transiently increased p70 S6 kinase in quiescent BHK GLP 2R cells, GLP 2 also inhibited extracellular signal regulated kinase 1 2 and reduced serum stimulated Elk 1 activity.
    Furthermore, no rise in intracellular calcium was observed following GLP 2 exposure in BHK GLP 2R cells.
    Although GLP 2 stimulated both cAMP accumulation and cell proliferation, 8 bromo cyclic AMP alone did not promote cell proliferation.
    These findings suggest that the GLP 2R may be coupled to activation of mitogenic signaling in heterologous cell types independent of **PKA** via as yet unidentified downstream mediators of GLP 2 action in vivo.

    - Brain Res Mol Brain Res, 1997   **Transcriptional activation of human choline acetyltransferase by AP2 and NGF induced factors.** .
      ChAT choline acetyltransferase is the enzyme responsible for acetylcholine synthesis and is specifically expressed in cholinergic neurons.
      To further characterize the transcriptional regulation of the hCHAT human ChAT gene by NGF, we examined the effects upon ChAT promoter activity of a family of transcription factors which are activated by NGF and several extracellular stimuli and encoded by immediate early genes.
      These include NGFI A Egr1, **zif268** , NGFI C Egr2 , Krox 20 and NGFI B Nurr77 .
      Two fragments of the hChAT gene were used for functional analysis carrying 944 bp P1 and 4000 bp P1 P2 of the 5 flanking region in front of the chloramphenicol acetyltransferase CAT reporter gene.
      They were transiently co transfected with NGFI A, NGFI C, Krox 20 and NGFI B expression vectors in NG108 15, SN6 and COS 1 cells.
      CAT activity after transfection of the p4000 ChAT CAT reporter into both neuronal cell lines NG108 15 and SN6 cells was increased up to 5 fold in the presence of co transfected NGFI A and up to 5 and 12 fold after co transfection of NGFI C expression vector in NG108 15 and SN6 cells, respectively.
      In NG108 15 cells, dbcAMP excerted a strong enhancing activity on the transactivation properties of NGFI C while this was not observed when cells were transfected with NGFI A.
      These trans activation effects were specific for neuronal cells.
      When NG108 15 cells were treated with dbcAMP in the presence of H89, a specific **PKA** inhibitor, the increase of transcriptional activity of NGFI C was abolished, indicating that a signalling transduction mechanism through **PKA** plays a role in NGFI C induced trans activation.
      Electrophoretic mobility shift assays showed that the sequence GCCCGGGGAG NGFRE located 1205 bp upstream of the first coding ATG E1 can bind NGFI A but not NGFI C.
      Several possibilities explaining the observed results are discussed.
      Finally, transfections of ChAT CAT reporters including the P1 P2 region or a minimal ChAT enhancer present in the P2 region in front of a heterologous promoter indicated the presence of a regulatory element which conferred AP2 dependent trans activation with homologous as well as with heterologous promoter constructs.

      - Biochim Biophys Acta, 1995   **Differential regulation of primary response gene expression in skeletal muscle cells through multiple signal transduction pathways.** .
        One of the earliest cellular responses to growth factors is the rapid induction of primary response genes.
        One group of such genes was originally isolated as tetradecanoyl phorbol acetate TPA inducible sequences TIS genes from mouse 3T3 cells.
        Proteins encoded by the TIS genes include two transcription factors TIS8 also known as egr1 NGFIA **zif268** and TIS1 also known as NGFIB nur77 N10 .
        We have examined the inducibility of these two genes in a skeletal muscle cell line in response to agents that have been reported to block muscle differentiation.
        We report here that basic fibroblast growth factor bFGF induced the expression of both TIS1 and TIS8 in mouse C2C12cells.
        Both genes were also inducible by TPA while forskolin which activates the cAMP dependent pathway induced TIS1 but not TIS8.
        Down regulation of protein kinase C PKC activity by TPA pretreatment repressed the bFGF induction of TIS1 but had little effect on the bFGF stimulated expression of TIS8.
        Moreover, while both TPA and bFGF stimulated the hyperphosphorylation of c RAF and the activity of MAP kinase, TPA pretreatment failed to block RAF phosphorylation or the stimulation of MAP kinase activity by bFGF.
        Induction of the two TIS genes in skeletal myoblasts therefore appeared to be dependent to different extents on the activation of protein kinase A **PKA** , PKC and MAP kinase.
